# Male Pedigree Toolbox: A Versatile Software for Y-STR Data Analyses

**DOI:** 10.3390/genes15020227

**Published:** 2024-02-10

**Authors:** Arwin Ralf, Bram van Wersch, Diego Montiel González, Manfred Kayser

**Affiliations:** Department of Genetic Identification, Erasmus MC, University Medical Center Rotterdam, 3015 CN Rotterdam, The Netherlands

**Keywords:** Y-STRs, male differentiation, automated data analysis, patrilineal genealogy

## Abstract

Y-chromosomal short tandem repeats (Y-STRs) are widely used in forensic, genealogical, and population genetics. With the recent increase in the number of rapidly mutating (RM) Y-STRs, an unprecedented level of male differentiation can be achieved, widening and improving the applications of Y-STRs in various fields, including forensics. The growing complexity of Y-STR data increases the need for automated data analyses, but dedicated software tools are scarce. To address this, we present the Male Pedigree Toolbox (MPT), a software tool for the automated analysis of Y-STR data in the context of patrilineal genealogical relationships. The MPT can estimate mutation rates and male relative differentiation rates from input Y-STR pedigree data. It can aid in determining ancestral haplotypes within a pedigree and visualize the genetic variation within pedigrees in all branches of family trees. Additionally, it can provide probabilistic classifications using machine learning, helping to establish or prove the structure of the pedigree and the level of relatedness between males, even for closely related individuals with highly similar haplotypes. The tool is flexible and easy to use and can be adjusted to any set of Y-STR markers by modifying the intuitive input file formats. We introduce the MPT software tool v1.0 and make it publicly available with the goal of encouraging and supporting forensic, genealogical, and other geneticists in utilizing the full potential of Y-STRs for both research purposes and practical applications, including criminal casework.

## 1. Introduction

With the recent increase in the number of rapidly mutating (RM) Y-STRs [1], an unprecedented level of genetic differentiation between related males can now be achieved [2]. RM Y-STRs can help in forensic casework where Y-STRs are the only available DNA evidence by excluding male relatives of the suspect as potential contributors to crime scene stains [3]. In addition, RM Y-STRs open up possibilities for new applications of Y-STRs in forensic genetics in solving patrilineal relationships to answer forensic questions such as via patrilineal familial searches or patrilineal investigative genetic genealogy [4]. Moreover, Y-STRs of different mutation rates including RM Y-STRs are useful for all types of genealogical research and application studies as they allow to confirm, establish, or correct male pedigrees [5]. Y-STRs are also used in human population genetic and genetic anthropology research for various purposes, typically considering unrelated males [6,7,8]. For instance, Y-STRs can provide information about the spatial and temporal origin and spread of Y-SNP based haplogroups [9,10]. Using Y-STRs of different mutation rates in principle allows for the investigation of events that occurred at different time scales. A recent study suggested that Y-STR mutation rates are influenced by the haplogroup and population background [11], which require further research through more Y-STR mutation rate studies in different populations that include Y-SNP haplogroup analyses.

Many studies on determining Y-STR mutation rates focused on father–son pairs [12,13,14,15], as this is considered the best approach for obtaining accurate mutation rate estimates for Y-STRs, as long as the number of pairs is large enough. However, large father–son pair studies are labor and resource intensive, as for every genetic transfer (meiosis) tested, two DNA samples need to be analyzed. Y-STR studies in pedigrees are less common but more efficient, as information about the same number of meioses can be obtained by analyzing less samples [16,17,18]. Moreover, they allow for the determining of male relative differentiation rates also beyond the very close relative pairs available in father–son pairs. Previous studies utilizing Y-STR analysis in pedigrees have focused on either shallow pedigrees, where males are separated by up to four generations, or deep-rooted pedigrees, where males are included that are separated by many more generations. Both types of pedigrees have their own advantages and limitations. In shallow pedigrees, the analysis and interpretation of Y-STR variations are relatively straightforward, but the number of meioses covered is modest, unless a large number of such pedigrees is included. Studying Y-STRs with relatively low mutation rates cannot be accomplished reliably when analyzing a low number of meioses. While deep-rooted pedigrees are more suitable for analyzing Y-STRs with low or medium mutation rates, for RM Y-STRs, they provide an increased risk of hidden mutations, which can negatively impact the accuracy of the mutation rate estimates obtained. However, a recent large study on Y-STRs with increased mutation rates involving both types of pedigrees [2] showed that the pedigree-based mutation rates were largely similar to the mutation rates previously established based on father–son pairs for the same Y-STRs.

Depending on the pedigree structure and complexity, the analysis and interpretation of Y-STR data can become challenging, especially when analyzing extended pedigrees and encountering many individuals for whom no genotypic data are available. Such pedigrees are often involved in genealogical research going back many generations. In forensic casework, large and complex male pedigrees can also be encountered such as in Y-STR-based mass screenings used to solve criminal cases that initially lack known suspects [19]. The manual analysis of Y-STR data can lead to uncertainty in the results and can limit the conclusions drawn from such analyses with an increased risk when the Y-STR data are larger and more complex. The development of novel software tools allowing the automated analysis of Y-STR data is needed especially for analyzing large and complex data, but such tools are scarce.

To aid Y-STR research and applications in different fields, forensics included, we introduce the Male Pedigree Toolbox (MPT), a user-friendly software tool for the automated analyses of Y-STR data in pedigree and population samples. By making the MPT software v1.0 publicly available, we encourage and support forensic, genealogical, and other researchers and practitioners to better appreciate the value that Y-STRs offer.

## 2. Materials and Methods

The Male Pedigree Toolbox (MPT) exists in two versions, a graphical user interface (GUI) and a command line tool. Both versions are available as executable files that can be run from both Windows and Linux. The GUI and command line interface can also be installed with the python pip command (pip install male-pedigree-toolbox). Two of the modules of the MPT, the simulation module and the prediction model-building module, are available only in the command line version. This is because both modules are highly computationally intensive and require a more powerful computational infrastructure than available with an average PC. Once the models are built, they can be applied to data in both the Windows and the command line version of the software v1.0. The minimum python version required to implement the tool is Python 3.6. The following packages have been used throughout the different modules. Numpy [20], pandas [21], scikit learn [22], scipy [23], matplotlib [24], tqdm [25], and PysimpleGUI (https://www.pysimplegui.org, accessed on 12 January 2024). The MPT was designed to work with data produced via capillary electrophoresis (CE); however, it could also be used on massively parallel sequencing (MPS) data, in which case, the MPS data would need to be transformed to a format that resembles CE data. Each of the following sections will describe one of the modules that was included in the MPT. A summary of the included models and the required files can also be found in Table A1. For the MPT to function correctly, all individual profiles need to have genotypic information for the same set of Y-STRs. In the consequence of if, for example, one sample has an incomplete profile lacking data for a single Y-STR, then either that Y-STR or that individual needs to be removed prior to the analysis.

### 2.1. Automatic Pairwise Distance Calculation from TGF Files

**Figure 1:**
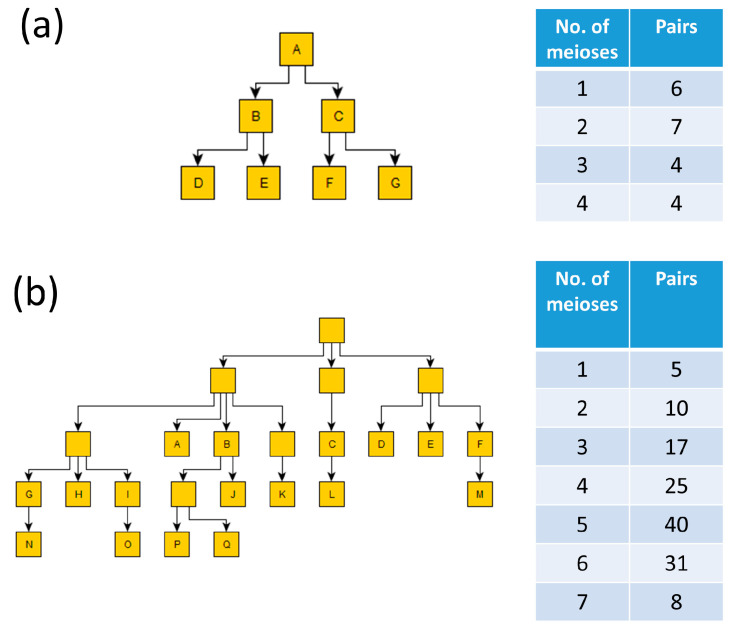
Two pedigrees and the corresponding number of pairwise meioses (e.g., 1 meiosis indicates a father–son pair, and 2 meioses indicates either a pair of brothers or a grandfather–grandson pair); the letters represent unique identifiers for individuals that have been genotyped: (**a**) a relatively small pedigree with all individuals included; and (**b**) a larger pedigree with some missing individuals (i.e., without genotype information), as shown by the lack of an identifier in the node.

Trivial Graph Format (TGF) is a format that can be used to depict male pedigrees; this format is used to provide known pedigree information in the MPT. It is a simple text format that can be used to visualize pedigrees using free software packages such as yEd Graph Editor (https://www.yworks.com/products/yed; accessed on 12 January 2024). The same software can also be used to draw complex male pedigrees and convert the structure to TGF. To perform analyses on known pedigrees, it is important to know the distance between each member of the given pedigree (i.e., the number of meioses separating them). With small pedigrees, this can be accomplished rather easily. For example, in a pedigree consisting of 7 individuals, as shown in Figure 1a, there clearly are 6 father–son pairs (1 meiosis), 4 grandfather–grandson pairs (2 meioses), 3 brother pairs (2 meioses), 4 uncle–nephew pairs (3 meioses), and 4 cousins (4 meioses). However, in larger pedigrees, as exemplified in Figure 1b, it would be more tedious to count all the pairs by hand. The first module of the MPT performs these calculations automatically. To do so, it differentiates nodes with and without a label. For this, it is assumed that from nodes with a label, there are genetic data available, while nodes without a label refer to individuals for whom no genetic data are present. The pairwise distance calculations are performed only between the individuals for whom data are available, while the individuals without available genetic data are considered exclusively for obtaining the correct meiotic distance determination.

### 2.2. Recognizing Pairwise Mutations

The second module of the MPT uses the Y-STR genotypic data to estimate the number of mutations that have occurred between any two individuals, purely based on the genotypes, i.e., without considering the underlying pedigree structure. This module of the MPT requires an input file containing the following for each Y-STR: an identifier for the pedigree, an identifier for the sample, the name of the Y-STR, and the observed Y-STR allele(s) (multiple in the case of multi-copy loci, or duplication events). Given the genotypes of each Y-STR in each pair of male relatives within a pedigree, the module will assess a conservative number of mutations required to explain the Y-STR variation in such a pair of samples (if any). In these calculations, the stepwise mutation model is assumed, in line with empirical data evidence from father–son pair studies showing that single-step Y-STR mutations are far more frequent than multi-step mutations [26]. For example, if in a given pedigree, individual 1 carries allele 15 for a specific single-copy Y-STR, while individual 2 carries allele 17 at the same locus, two single step mutations are assumed rather than one two-step mutation.

At multi-copy loci, these calculations are typically less straightforward than the example given above. Therefore, in order to determine the minimum number of Y-STR mutations that can explain variations between two individuals at multi-copy loci, we used a difference matrix. This matrix puts one individual along each axis and determines the minimum number of mutations required to explain the variation of each allele between both individuals. The preferred solution for the matrix is then determined as the minimum path over this matrix using minimum weight matching in bipartite graphs. In order to allow the minimum path calculation, the number of copies between individuals needs to be harmonized. This means that it needs to be determined which loci are duplicated, since it is unknown how many copies each allele has in the multi-copy locus. For this purpose, the genotypes of all individuals assigned to the same pedigree are taken into account; the individual with the most copies will determine the number of copies that are assumed. The rationale that was applied behind these calculations for the more complex multi-copy Y-STRs has been more extensively described elsewhere [2].

When the output file from the automatic pairwise distance calculation (see Section 2.1) is provided, the pairwise mutation module will additionally calculate the male relative differentiation rate (i.e., the number of pairs that show at least one mutation relative to the total number of pairs that were considered) for each meiotic distance and over all pedigrees included in the analysis. Additionally, the Clopper–Pearson 95% binomial confidence intervals for the estimated differentiation rates will be calculated. This module can also automatically produce a file that serves as an input to the prediction module that will be described in Section 2.7.

Lastly, the output from this module can be applied to unrelated individuals in population genetics as an alternative way to measure haplotype diversity. The median number of pairwise differences (Rst) could then be used as a measure of diversity. This method allows for comparisons of differences between Y-STR marker sets or different populations, even in the absence of shared haplotypes, as demonstrated in a previous study [11].

### 2.3. Estimating Y-STR Mutation Rates from Pedigrees

The MPT v1.0 allows for the estimation of Y-STR mutation rates in a locus-specific manner. To perform this analysis, the software requires an input file containing the Y-STR genotype information (as described in Section 2.2), and TGF files containing the pedigree structures (as described in Section 2.1). From the TGF file, this module first calculates per pedigree the total number of meioses covered by all pairs of male individuals in the pedigree of which genotypic data are available. Secondly, it will calculate per Y-STR locus the most likely number of mutations that had occurred in the pedigree in the following way. In cases where an allelic difference is seen between a father and his son, it will be regarded as a single mutation no matter how many repeats are found to be different. For example, an allele 10 to 12 mutation in a father–son pair is regarded as one two-step mutation because both are separated by only one meiosis. In contrast, when such allelic differences are observed in a pair of cousins, separated by 4 meioses, and in the absence of genetic data from at least one of their fathers, two independent single-step mutations are assumed in line with the stepwise mutation model.

By utilizing TGF files, the module redraws the pedigree structure using Graphviz [27] and identifies the specific Y-STR locus where a mutation was placed in order to explain the observed genotype variation in accordance with the pedigree structure. This module too always strives to explain the data with as little mutations as possible following the stepwise mutation model. Additionally, it will produce a drawing of the pedigree highlighting all mutations observed using all Y-STRs. Individuals will be colored accordingly, directly visualizing how different clusters of individuals can be differentiated from each other within the pedigree. These visualizations can be used to infer the most likely founder haplotype of the given pedigree, which will be depicted in white in this drawing.

### 2.4. Drawing Dendrograms

The output file containing the Y-STR genotypic variation per-locus for all the pairs of males in a given pedigree described in Section 2.2 serves as input file for the dendrogram module. Using all pairwise differences observed in each pedigree, a dendrogram is drawn. Additionally, the module has the option to provide marker-specific mutation rates, which can be used to assign a weight to each observed variation. Here, Y-STRs with increased mutation rate carry less weight than those with reduced rates. This weight is defined as the –log10 of the mutation rate. By default, the clustering divides each dendrogram into the optimal number of clusters based on the silhouette score based on the algorithm provided by scikit learn [22] using hierarchical clustering with complete linkage. The user can, if desired, choose a specific number of clusters to be distinguished within the pedigree. The dendrograms are drawn using matplotlib [24].

### 2.5. Simulating Relative Pairs

This module is intended to produce training data for the prediction model, as described in the next sections. However, it may also prove useful for other purposes where large, simulated datasets can provide new insights into the dynamics of stochastic processes like Y-STR mutability. Using mutations rates (provided by the user), the simulation module generates a user-defined number of pairs in a user-defined number of separating meioses. For every multi-copy marker, each locus is defined separately with its locus-specific mutation rate equal to the total mutation rate of the multi-copy locus divided by the number of loci. Multi-copy loci have to be indicated with _a–z, for example, DYF399S1 has three copies and would receive three separate entries named DYF399S1_a, DYF399S1_b, and DYF399S1_c. In the simulation output file, all mutations observed at a locus will be combined to yield the total per-marker number of mutations for each multi-copy locus in each simulated pair. On top of the name of the loci, the input file includes the probability of not observing a mutation (i.e., 1-mutation rate), and the probabilities of observing a 1-step mutation and a 2-step mutation, respectively. In the pre-computed models, a 2-step mutation of 0.03 × the total mutation rate is used; hence, the probability for a single-step mutation would become 0.97 × the total mutation rate. For the DYF1000_a–d locus, an exception is made as it contains both a trinucleotide and a hexanucleotide repeat. As a result, it is not possible to distinguish between multi-step mutations at the trinucleotide repeat and single-step mutations at the hexanucleotide repeat. Therefore, at this specific locus, the 2-step mutation rate is set to 0.06 × the total mutation rate.

When the simulation starts, the value 0 is assigned to each of the Y-STRs; this is the ground state, meaning that no mutation has occurred. The probability of observing 0, 1, or 2 mutational steps in the first simulated generation is equal to the previously described user-defined probabilities. Importantly, the numbers do not indicate a forward mutation, but rather that a single mutation has occurred that could have been either in the forward or in the reverse direction. Therefore, negative numbers cannot occur in the simulation; each number simply indicates the number of steps that the allele deviates from the ground state. For the following generations, if the value remained 0 in the previously simulated generation, the probabilities remain the same. However, if the previous generation led to a mutation, the probabilities will be halved and become directional. For example, from state 1, it is assumed that there is an equal probability of mutating backward to state 0 as of mutating forward to state 2. Each generation is simulated independently, i.e., when simulating generation 2, instead of using the already simulated data from generation 1, a new temporary generation 1 is simulated from which a second-step generation 2 is simulated, the data from the temporary generations are not stored.

### 2.6. Building New Models with Machine Learning

The MPT also allows for the building of new machine learning models to predict the degree of relatedness of a given pair of relatives based on simulated data. The module is a wrapper around many different scikit learn classification machine learning modules [22]. Each model has a pre-defined parameter space for all or most of the configurable model parameters. Users then have the ability to choose one or more of these models in order to create classifiers based on simulated data. It is advised to apply at least 1000 simulated pairs per generation. While a higher number tends to result in slightly more accurate models, it can lead to a vast increase in running time. Models are created using double cross-validation since a large parameter space has to be searched. First, the input data are split into K definable folds, where each fold is used for testing the models and the remaining folds are used for training the models. Secondly, skikit learn’s RandomizedSearchCV is used to draw J definable random combinations of parameters used to create the models. This means that each model is trained on each subset of the data with a large variation in the parameters chosen. At the end, the top performing model for each fold K is chosen using the best estimator variable of RandomizedSearchCV. The F1 score, accuracy, precision, recall and Mathew’s correlation coefficient are all calculated for these classifiers and saved in a table. Additionally, a confusion matrix is drawn to provide an indication of the performances of the models.

Pre-computed models for the Y-STRs included in the most frequently used commercial and non-commercial Y-STR kits (e.g., Powerplex Y23, Yfiler Plus, and RMplex) based on recent mutation rate estimates [12] are provided together with the executables when installing the toolbox. These models have also been implemented on the website: https://ystr.erasmusmc.nl (accessed on 12 January 2024). More detailed information about these pre-computed models has been provided elsewhere [2].

### 2.7. Running the Prediction Models

To run a prediction model, a single input file is required. An option to generate this file automatically is included in the module described in Section 2.2. In the case of using pre-computed models, based on the observed variations in any pair of males, it will assign probabilities to each meiotic distance in a range from 1 to 50 meioses. In using the per-generation probabilities, the most likely ranges with 85%, 95%, and 99% confidence are calculated, as was previously described elsewhere [2]. The probabilities can be visualized by plotting; it has to be noted though that when performing large numbers of comparisons, the processing time will increase significantly by including these plots.

## 3. Discussion and Examples

The MPT v1.0 is a versatile software tool for analyzing and interpreting Y-STR data in male pedigrees of all types and in various scenarios. It is ideal for research purposes, as it can automatically analyze data from large numbers of pedigrees, including those with close relatives, such as father–son pairs, as well as pairs of distantly related males. The MPT is suitable for highly complex pedigrees, including those with many individuals, and can perform these analyses quickly and systematically. The prediction-based modules of the MPT are particularly useful for forensic casework and genealogy when the pedigree structure or the identity of a crime scene stain’s donor is unknown. However, the software also has some limitations. For instance, it is not able to process samples with incomplete data; therefore, if a sample has missing data, either that sample or the loci with missing data need to be excluded prior to the analysis. Moreover, currently, the foreseen application of the software is in a research setting, or to be used as guidance in forensic investigations. While some elements of the software hold potential to be used in court, these will have to be accompanied according to rigid statistical analyses and thorough validation, which is beyond the scope of the current study. Furthermore, it has to be noted that mutation rate analyses based on pedigrees with incomplete genotyping data (i.e., individuals in the pedigree that were not tested) rely to a certain degree on assumptions and thus can result in erroneous interpretations. That being said, the same would apply when performing the analysis manually on the same pedigree.

The following sections show various examples of how the MPT can be used. It is important to note that the final outcome will be influenced by factors such as the Y-STR set being used, the pedigree structure, and the population of the individuals. However, these examples demonstrate the capabilities of the MPT.

### 3.1. Examples for Dendrograms from Pedigree and Population Data

**Figure 2:**
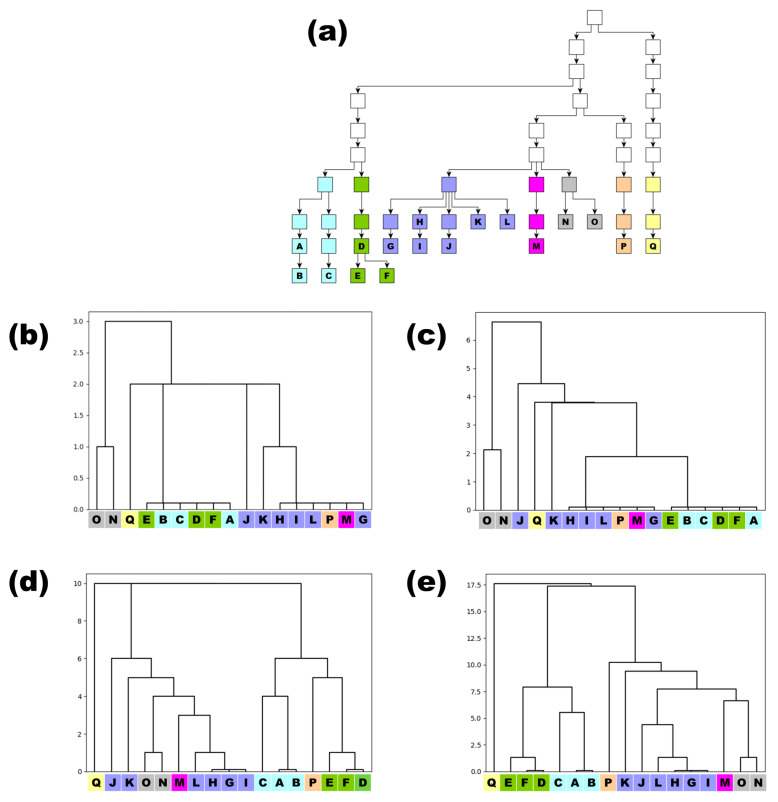
Pedigrees of male individuals with known patrilineal relationships (coloring was added for illustrative purposes and not part of the MPT): (**a**) the actual pedigree structure; (**b**) the substructure depicted as dendrograms drawn through the use of the MPT solely using Y-STR haplotypes based on Yfiler Plus without applying weights; (**c**) the analysis of the same data when weight is applied; (**d**) the dendrogram based on RMplex data without applying weight; and (**e**) the RMplex data while applying weight. The numbers on the Y-axis of Figure 2b–e represent the distance between the individuals, which is equal to the total number of observed variations (unweighted), or the sum of the number of variations per Y-STR multiplied with the −log10 of the mutation rate of the same Y-STR (weighted).

As explained previously, the pairwise distances obtained from the pairwise mutation module of the MPT can be used to construct dendrograms that are purely based on the Y-STR haplotype differences between the individuals in the defined pedigree. Figure 2a shows an example pedigree containing 17 males genotyped with Y-STRs. The most recent common ancestor (mrca) dates back nine generations from the most recent generation included in the pedigree. The coloring is based on the males six generations after the mrca. The 17 individuals were genotyped with both Yfiler Plus and RMplex, and the haplotypic differences for both assays were determined using the pairwise mutation module. Figure 2b,c show dendrograms obtained from the Yfiler Plus data without and with weights being applied, respectively. Figure 2d,e show the dendrograms based on the RMplex data. These examples clearly demonstrate the added value that RM Y-STRs provide relative to Yfiler Plus, as deeper substructures in the genotypic data could be visualized based on RMplex data. Moreover, at least in this example, it is evident that applying the weight approach leads to improved results. The rationale behind applying weights is that in Y-STRs with relatively high mutation rates, the same mutation may occur more than once independently within the same pedigree, making such Y-STRs less suitable to determine the fundamental structure of the dendrogram. On the other hand, Y-STRs with low mutation rates will only rarely mutate within a pedigree; if such a mutation does occur, it will therefore be assumed to be highly informative to determine the fundamental structure. Noteworthy, the pedigree structure shown in Figure 2e, which is purely based on the Y-STR data, shows a very high similarity to the pedigree structure known from records, as shown in Figure 2a. This notion highlights the potential benefit when attempting to identify an unknown suspect while having only identified some of his (distant) relatives through Y-STR haplotype similarities. Obviously, the success of this approach is subjected to the stochastic effects of Y-STR mutations that occurred within pedigrees. For example, a low number of mutations, individuals that underwent the same mutation independently, or a remarkably high number of mutations in certain branches can result in the formation of substructures in the dendrograms that do not necessarily reflect the true pedigree structure. Nevertheless, this form of automated pedigree reconstruction can be highly useful in forensic casework, particularly when complex pedigrees are encountered. In particular, if, in the future, Y-STRs were to be stored in national offender DNA databases, using RM Y-STRs in this manner may be an efficient method for identifying unknown perpetrators.

**Figure 3:**
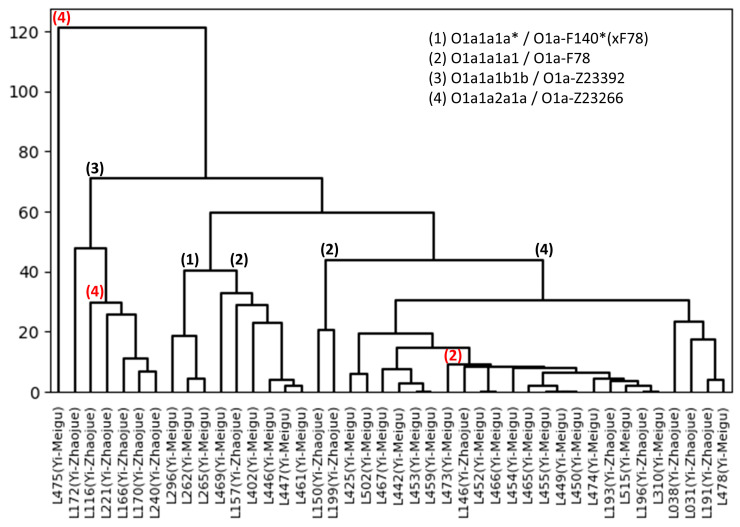
A dendrogram obtained with the MPT based on Y-filer Plus population data from Wang et al. 2021 [28] showing a subset of 42 males belonging to different subhaplogroups of the Y-SNP haplogroup O1a. Note the strong concordance between the Y-STR-based dendrogram and the phylogenetic Y-SNP subhaplogroup tree indicated by branch numbers. Red numbers indicate remarkable deviations between the Y-STR-based dendrogram and the Y-SNP phylogeny.

**Figure A1:**
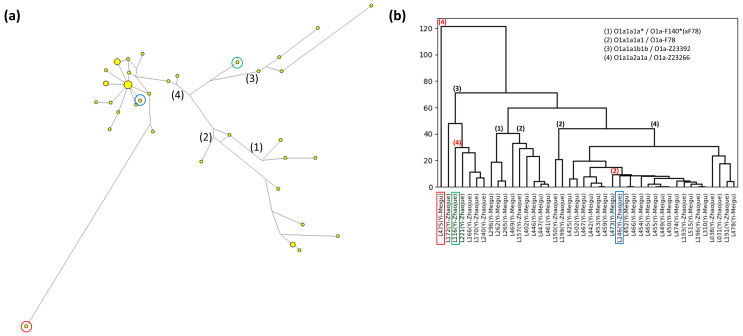
The dataset from Wang et al. (2021) [28] with outliers highlighted by the same colors and haplogroups clusters depicted by numbers: (**a**) visualized as a median-joining network; and (**b**) visualized as a dendrogram using the MPT.

Besides applications on pedigrees containing relatively, closely related men, the dendrogram module can also be used in population genetics typically involving unrelated individuals. To exemplify, we used a part of the supplementary data from Wang et al. 2021 [28]. This study typed their population samples with both Y-STRs, specifically Yfiler Plus, and with 193 Y-SNPs. Here, we retrieved the Y-STR genotypes of 42 males typed with Y-SNPs as belonging to various subgroups of haplogroup O1a. Using the pairwise mutation module, all haplotypes were compared to each other. Based on the obtained number of differences between each pair, a dendrogram was drawn from the Yfiler Plus data while applying weights. As shown in Figure 3, based on this Y-STR analysis, the majority of samples cluster according to their Y-SNP-defined subhaplogroup. Additionally, substructures within subhaplogroups became apparent, for example, samples L150 and L199 branching off from the other O1a-F78 individuals. Such substructure may be of use when searching for additional Y-SNPs to further dissect the subhaplogroups. Remarkably, some individuals in Figure 3 appear to cluster with the wrong subhaplogroups (i.e., samples L116 and L473). It is possible that these individuals are outliers and that the observations, therefore, reflect the true Y-chromosome genetics. However, it is also possible that errors or mix-ups were introduced in either the Y-STR or Y-SNP typing. Therefore, repeating both analyses in such cases would be advisable. Additional scrutiny may also be required when observing individuals that fail to cluster with any other sample within the same broad haplogroup (i.e., sample L475 in Figure 3). In such cases the haplotype appears to be highly divergent, which may indicate a different evolutionary origin of the male lineage (i.e., falling in a different subhaplogroup). In summary, the use of dendrograms in population genetics can aid in the detection of substructures within haplogroups and populations, as well as in data quality control. This approach could serve as an alternative for using median-joining networks (MJNs) [29], or both approaches could be combined and compared. An example of an MJN generated using Network 10.2.0.0 (fluxus-engineering.com, accessed on 12 January 2024) on the same data is shown in Figure A1. Overall, the genetic substructures visualized by the dendrograms and the MJN correspond well; the outliers that were observed with the dendrograms also appear to be outliers in the MJN. An advantage of the dendrogram from the MPT is that the multi-copy loci can also be included in the analysis; for the MJN, these Y-STRs had to be excluded.

### 3.2. Example for Mutation Analysis

Accurate mutation rate estimates of Y-STRs are important in forensic genetics, particularly when more sophisticated methods for calculating the evidential value of a match are pursued [30,31,32]. When attempting to estimate mutation rates using pedigree data, uncertainty exists depending on the complexity of the pedigree structure and the number of individuals without genetic data available. Within the MPT, we implemented a systematic way to deal with such cases. This method may not always deliver 100% truthful interpretations; rather, it selects an interpretation that fits with the data and requires the least number of mutational steps. Thus, it provides a conservative, systematic interpretation. In contrast to the pairwise mutation module that can be used to create dendrograms using only genetic data, the pedigree mutation module also requires information about the pedigree structure.

**Figure 4:**
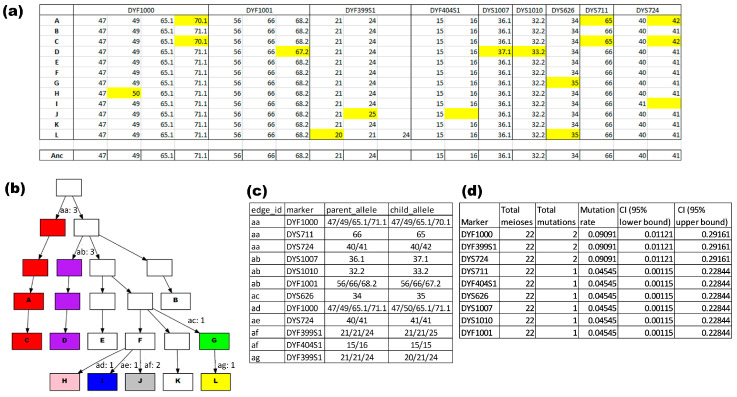
Mutation rate analysis performed by the MPT: (**a**) the genotypic input of the Y-STRs that were variable within this pedigree; mutations are highlighted in yellow; (**b**) the visible output where the MPT draws the pedigree and indicates mutations and uses coloring to show unique haplotypes within the pedigree; (**c**) the specifications of the mutations; and (**d**) the format of the output table used to estimate the mutation rate.

An example of the mutation rate module applied on a single pedigree is shown in Figure 4. Figure 4a shows a total of nine Y-STRs that were variable within the 12 genotyped males of the pedigree. Combining this specific pattern of mutations with the known pedigree structure resulted in the three output files shown in Figure 4b–d. Figure 4b shows a drawing of the pedigree structure; the labels at the edges refer to specific (sets of) mutations, which are specified in a separate file (Figure 4c). The colors in the pedigree drawn in Figure 4b indicate unique haplotypes. In this example pedigree, the ancestral haplotype (always shown in white), which is the haplotype putatively carried by the mrca of these males, is found in four of the genotyped males. However, in reality, the mrca haplotype might have held one of the variant alleles from the individual A/C lineage, and the ancestral haplotype shared by the four individuals in this pedigree could have arisen from a mutation happening one generation later. Similarly, each of the three mutations that set individual A and C apart from all other individuals within this pedigree could have occurred either one, two, or three (i.e., in sample A) generations after the mrca. For simplicity, the output will always show the mutations as high up in the family tree as possible.

Regardless of where exactly in the pedigree the mutations occurred in reality, given the variant data, it is certain that these mutations must have occurred somewhere in the lineages. Therefore, the exact position where the mutations are placed does not interfere with accurate mutation rate estimation. However, there are scenarios where mutations could be missed, which is a limitation that is inherent to mutation analysis using pedigrees. For example, if the same mutation occurred independently in two parallel branches, the software may incorrectly interpret it as a single mutation in a common ancestor. Or worse, the mutation may be completely obscured, depending on the number of individuals typed and the structure of the pedigree. Such uncertainties, however, only exist in pedigrees that include individuals for which no genotypic data are available. Moreover, a manual interpretation of the data would likely lead to the same erroneous interpretation.

Figure 4d summarizes the mutation rate outcome for this specific pedigree: the pedigree covers a total of 22 meioses, and for all Y-STRs, the outcome was either 0 (only one of such Y-STR is shown), 1, or 2 mutations. Of course, with such a low number of meioses, the mutation rates are highly unreliable, as indicated by the 95% confidence intervals. However, when analyzing many separate pedigrees, the MPT will provide the overall mutation rate estimation by combining the results obtained from all pedigrees. That such rates are highly similar to those obtained when analyzing only father–son pairs was shown in a previous study that had already used (an early stage in-house version of) the MPT for this purpose [2].

### 3.3. Simulating Pedigrees and Predicting the Level of Relatedness

In forensic casework, it may occur that two similar Y-STR haplotypes are observed. If one such a haplotype is derived from a known male while the other male remains unknown, this similarity may provide a lead for further investigation. In particular, when Y-STRs with high mutation rates still result in a match, this may indicate that both males could be closely related. Several methods for estimating the time since the most recent common ancestor have been proposed based on Y-STR haplotypes [33,34]. Recently, a new approach based on simulation and machine learning was introduced to estimate the relatedness of the donors of two similar Y-STRs haplotypes. The precision and accuracy of the approach have already been well documented in previous research [2]. Several precomputed models can already be used through a web interface at https://ystr.erasmusmc.nl (accessed 12 January 2024). However, only a limited number of models were generated to be accessible online. The MPT provides the opportunity to build new prediction models, for example, on a specific new set of Y-STRs, or using population-specific mutation rates. Also, when a partial Y-STR profile is obtained, it may be of interest to build a new model including only the Y-STRs of which genotypic data were obtained. The precomputed models span a range of 1 to 50 meioses; however, it may be desirable in specific cases to look to a broader or a narrower range. The simulation and prediction model-building modules of the MPT offer users full flexibility to build the models that are most suitable for their research needs.

**Figure 5:**
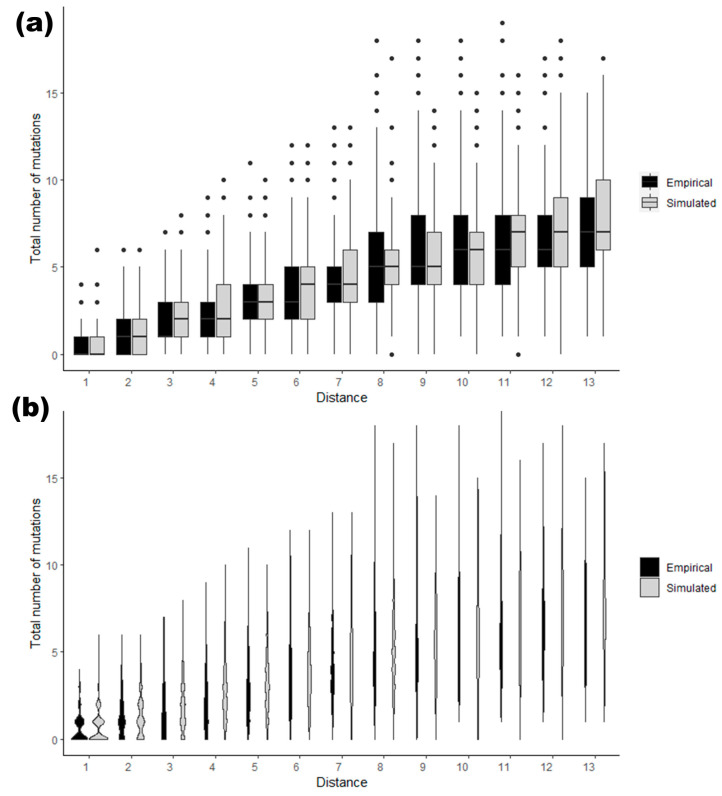
Comparing the distributions of the number of variations observed between pairs of males in empirical and simulated data in a meiotic distance range of 1 to 13: (**a**) shown as boxplots; and (**b**) the same distributions shown as violin plots.

It is of importance that the simulated data used for training the model are comparable to the actual haplotypic variation that is found in a pedigree. Figure 5 shows that, indeed, this was the case in this example. Here, we conducted a simulation of 1000 pairs of male relatives using the consensus mutation rates from a previous study [12]. The simulation covered a range of meiotic distances from 1 to 13, and for each pair, we plotted the number of Y-STR differences observed between the members. We also plotted empirical data from a previous publication [2] showing the distribution of number of Y-STR differences observed within real pedigrees. As evident from Figure 5, the distribution of the number of Y-STR differences between the simulated and empirical datasets in this example are indeed highly similar. Thus, fulfilling the requirement for applying a model that was trained based on simulation data on empirical data. However, new evidence suggests that more complex patterns of Y-STR mutations are in play [35,36]. Once more comprehensively established, incorporating such features could potentially further refine the simulation and model-building in the future.

## 4. Conclusions

We herewith introduce the MPT v 1.0, a user-friendly software tool for multi-purpose automated analyses and interpretations of Y-STR data in male pedigrees of all types and complexity, which we make publicly available for widespread application. We expect the MPT to become widely applied for both research and application purposes in both forensic and genealogical genetics as well as in the interplay between both for patrilineal investigative genetic genealogy.

## Data Availability

The Male Pedigree Toolbox (MPT) can be obtained from https://github.com/genid (accessed 12 January 2024) together with the documentation on the installation of the different versions on different operating systems, documentation on the usage of the software, and example data.

## Appendix A

**Table A1:** Concise descriptions of the different modules included in the Male Pedigree Toolbox and the associated input and output files.

Module Name	Input File(s)	Output File(s)	Purpose
1. Pairwise distance	- TGF file(s) in a folder	distances.csv	Calculating the number of separating meioses between all individuals with genotypic data in the pedigree
2. Pairwise mutation	- Allele file (csv) - (distances.csv)	- Summary_out.csv - Full_out.csv - (Differentiation_out.csv) - (Prediction_out.csv)	Comparing the genotypes of all typed individuals in the pedigree and calculating the number of mutational steps between each individual. Calculating the differentiation rate and making a prediction file are optional.
3. Pedigree mutation	- Allele file (csv) - TGF file(s) in a folder	*Per pedigree:* - Pedigree drawings of all markers and markers with mutation individually (pdf) - all_marker_edge_info.csv - mutation rate within pedigree (csv) *Overall pedigrees:* - Total_mutations.csv	Performing a mutational analysis, taking the provided pedigree structure into account. Visualizations are an easy way to view genetic variation within the pedigree. When typing multiple pedigrees, the overall mutation rate can be automatically estimated using this module.
4. Dendrograms	- Full_out.csv - (Mutation rate file (csv))	*Per pedigree:* - A dendrogram (png)	Generating dendrograms that can be used to create visualizations of genetic similarities when the pedigree structure is (partially) unknown. By providing a file with the mutation rates, the distances will be weighted where markers with a higher mutation rate have less influence.
5. Simulate (command line only)	- Mutation rate file (2stepmodel (csv))	- Simulation_output (csv)	Based on the provided mutation rates, a user-defined number of simulations will be performed on a user-defined number of separating meioses. These data can be used as input for prediction models but can also provide insight about the expected mutational behavior of a given set of Y-STRs.
6. Model builder (command line only)	- Simulation output (csv)	- Model output (csv)	Here, users can build their own custom models; this may be required, for example, when using a non-standard Y-STR kit, or when the mutation rate in the population of interest deviates strongly from the mean rates.
7. Running Prediction	- Prediction file - Model file - (Model training file)	- Prediction table - (Prediction plots)	If the user wants to validate their prediction model using empirical data, this module allows for a large number of predictions to be conducted using a pre-computed or a custom prediction model.
